# Additional Support for Simple Imputation of Missing Quality of Life Data in Nursing Research

**DOI:** 10.5402/2011/752320

**Published:** 2011-11-16

**Authors:** Wilma M. Hopman, Margaret B. Harrison, Meg Carley, Elizabeth G. VanDenKerkhof

**Affiliations:** ^1^Clinical Research Centre, Kingston General Hospital, Kingston, ON, K7L 2V7, Canada; ^2^Department of Community Health and Epidemiology, Queen's University, Kingston, ON, K7L 2V7, Canada; ^3^School of Nursing, Faculty of Health Sciences, Queen's University, Kingston, ON, K7L 2V7, Canada; ^4^Practice and Research in Nursing (PRN) Group, Queen's University, Kingston, ON, K7L 2V7, Canada; ^5^Department of Anesthesiology and Perioperative Medicine, Queen's University, Kingston, ON, K7L 2V7, Canada

## Abstract

*Background*. Missing data are a significant problem in health-related quality of life (HRQOL) research. We evaluated two imputation approaches: missing data estimation (MDE) and assignment of mean score (AMS). *Methods*. HRQOL data were collected using the Medical Outcomes Trust SF-12. Missing data were estimated using both approaches, summary statistics were produced for both, and results were compared using intraclass correlations (ICC). *Results*. Missing data were imputed for 21 participants. Mean values were similar, with ICC >.99 within both the Physical Component Summary and the Mental Component Summary when comparing the two methodologies. When imputed data were added into the full study sample, mean scores were identical regardless of methodology. *Conclusion*. Results support the use of a practical and simple imputation strategy of replacing missing values with the mean of the sample in cross-sectional studies when less than half of the required items of the SF-12 components are missing.

## 1. Introduction

Health-related quality of life (HRQOL) is an increasingly important outcome in both clinical trials and observational studies [1–4]. It is also a natural choice as an outcome for nursing interventions since interventions are often aimed at improving well-being. This is particularly true in chronic disease management, where a cure does not exist and the goal of treatment is often to optimize comfort, and learn to live with and manage one's condition [1, 3, 4].

Missing data are a significant problem in HRQOL research due to the potential loss of statistical power as the sample size is reduced and, more importantly, due to the potential for bias [5, 6]. For example, if those who are sicker are less likely to complete the assessment, HRQOL based on those with complete data may be overestimated; conversely, if those who are feeling well drop out of a study, HRQOL may be underestimated [5]. Yet missing data continue to be an issue, even when specific interventions to minimize missing data are used [7].

The potential impact of the problem has been well described [5], including the impact of data that are missing completely at random (MCAR), missing at random (MAR), or missing not at random (MNAR). A number of researcher teams have developed strategies for imputation of missing data, including modified regression estimation [8], missing data estimation (MDE) [9], single and multiple imputation strategies [10], regression-based multipattern imputation [11], last value carried forward/next value carried backward approaches in longitudinal data [12], and hot deck techniques [13].

The Medical Outcomes Trust SF-36 and the shorter SF-12 health surveys are the most widely used HRQOL assessments in the world, with translations into 138 and 113 languages, respectively [14]. When scored, the SF-12 produces a Physical Component Summary (PCS) and a Mental Component Summary (MCS) [15]. All 12 items contribute to the two components, but six are primarily used to generate the PCS while the other six are primarily used for the MCS. The problem is that if even one of the twelve items is missing, the PCS and the MCS are not produced. This can lead to nonresponse bias and is a particular issue for those who lack the funds to purchase commercial applications for missing data estimation, or for routine users for whom the increase in computational complexity associated with multiple imputation strategies puts such approaches out of reach [6].

One practical approach to this problem has been put forward by Perneger and Burnand [6]. They have provided a simple approach to use when fewer than four of the six key items of the PCS or the MCS are missing, which involves replacing the missing values with the mean of the population under study. They found that using this publically available strategy significantly reduced the amount of missing data, while still providing satisfactory results; the mean intraclass correlation (ICC) between the imputed and true score was 0.979 for both the PCS and MCS [6].

Perneger and Burnand were able to test the difference between the imputed and the true score; however, in reality, the reason for imputation is that the true score is not available. In a recent randomized clinical trial of two approaches to compression bandaging for leg ulcers [16], small amounts of missing data prompted the study team to utilize MDE [9], a proprietary program with associated costs. Using this methodology, missing data are estimated using algorithms that consider the pattern of responses across available items or, if only one item is available, the relationship of that item's response categories to the total score [9, 12].

We sought to compare the PCS and MCS scores generated using MDE [9] with those derived using assignment of mean score (AMS) [6] and to compare the effect on the overall study results using both methodologies.

## 2. Methods

SF-12v2 [15] was collected as part of a larger Canadian multicenter randomized controlled trial comparing two types of compression bandaging technologies [16]. Community care clients presenting with venous leg ulcers were screened for eligibility and the appropriateness of management with high compression therapy. Eligible, consenting individuals were randomly allocated to one of the two technologies. The primary outcome was time to healing and secondary outcomes included self-reported measures including HRQOL. Data from the *cross-sectional* baseline administration of the SF-12 are reported here. The survey was either completed by the participant independently during their regularly scheduled nursing care visit or completed with the help of a caregiver. Ethics approval for the RCT was granted by the Queen's University Research Ethics Board (REB# NURS-140-03), and local approval was granted at individual sites where required.

Study personnel were licensed to use the MDE [9] to estimate missing data and produce the PCS and the MCS on the basis of these estimated data. However, in a parallel process, the missing data were also estimated by assigning the mean score of the sample, and the PCS and MCS were recalculated using the published scoring algorithms. Summary statistics were produced for the component summaries using each methodology and compared using ICC. Differences between the assigned scores were also calculated. Scores were then included in the larger sample to assess the impact of the imputed data on the overall sample summary statistics.

## 3. Results

Complete SF-12 data were available for 384 (90.6%) of the 424 participants. However, 23 (5.4%) were missing one or more items and 17 (4.0%) missed the entire questionnaire. These 17 were not included in the imputation by MDE or AMS. Two of the 23 were not included since one had too much additional information missing for MDE assignment and the other was randomized after MDE had been completed.

For the remaining 21 respondents, 20 missed 1 item and 1 missed 2 items for a total of 22 missing items. Of these, most were in the physically oriented domains (16/22). The first general health item (gh01) was missed twice; each of the physical function items (pf02, pf04) was missed 5 times; the first role physical item (rp02) was missed 4 times. For the mentally oriented items, the second role emotional item (re03) was missed once, the first mental health item (mh03) was missed twice, and the vitality item (vt02) was missed 3 times. This suggests that individual items, rather than entire sections, were missed; the one person who missed two items missed a physical function and a mental health item.

**Figure 1:**
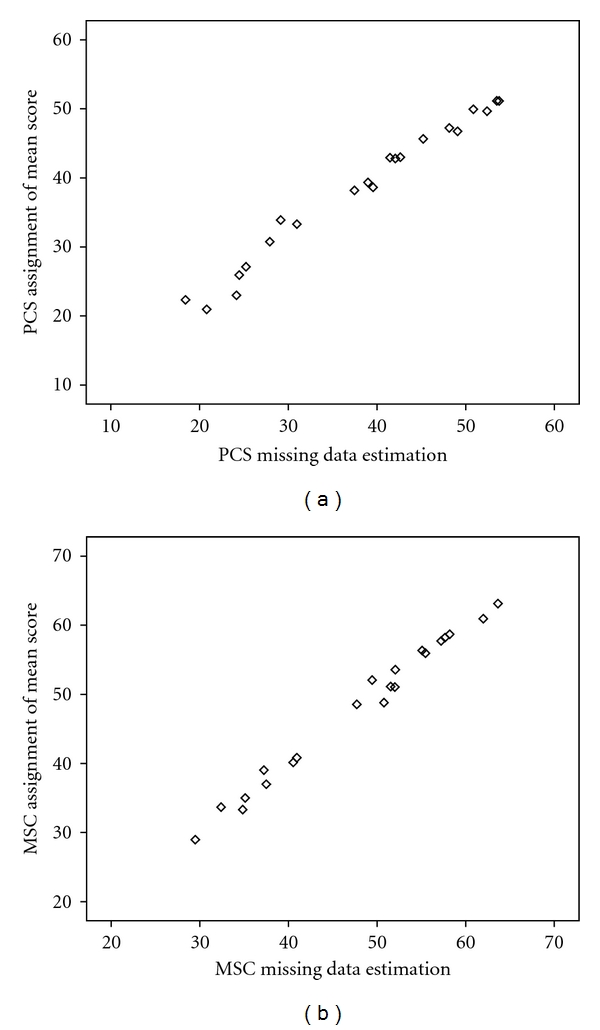
Scatterplots comparing PCS and MCS scores derived using the missing data estimation and assignment of mean score approaches.

Figure 1 contains a scatterplot for the PCS and MCS values derived using the MDE and AMS methodology.  Table 1 contains the mean and standard deviation (SD) for the two imputation methods, the difference between the values, and the overall sample mean with and without the imputed data. When subtracting the MDE-derived values from the AMS-derived values, the means of the change scores were positive, suggesting that overall results were slightly higher for the AMS values. For the PCS, the range of change scores was −2.74 to 4.74 (mean 0.35 and SD 2.09), while for the MCS the values were −1.98 to 2.62 (mean 0.16 and SD 1.14). While there is some variability in individual assignments, mean values are very similar, with ICCs >  .99 for both the PCS and the MCS. When the imputed data were added into the larger sample, the method of imputation had no impact, as the mean scores and 3 of 4 SDs were identical. The SD for the PCS differed by a negligible 0.1 points (10.0 for MDE and 9.9 for AMS).

## 4. Discussion

These data support the use of a practical and simple imputation strategy of replacing missing values with the mean of the population under study when less than half of the required key items of the SF-12 components are missing in cross-sectional research. This approach is not suitable for cases where the entire questionnaire is missing, where there is a clear pattern of missing data, or for data missing longitudinally, where more advanced statistical methodology should be utilized [5, 8–12]. Moreover, the approach is also suboptimal when inferences are to be made about individuals [6]. Despite the fact that the differences in the mean values were minimal, there were occasional large differences in individual estimates (Figure 1).

Application of the group mean for an individual item will result in some regression towards the mean, particularly for those with extreme scores [5, 6]. This is less of a problem when an occasional item is missed, as compared to situations where the entire instrument is missed. Even so, both the current findings and previous research [6] suggest that those with missing data do have slightly lower HRQOL scores. This was particularly apparent for the MCS scores, where those with imputed data scored close to 3 points lower on the MCS than those with complete data; the comparable difference for the PCS was closer to one point.

This study is limited by the small sample size, by the fact that participants are from a single study, and most had only 1 item missing; it is possible that the comparison might have turned out differently had many been missing 2-3 items. However, the results are not intended to be definitive; rather, they are intended to provide additional support for the larger study which proposed this methodology [6].

Obviously the best approach to missing data is to prevent it by making every attempt to obtain complete data. However, even the most carefully designed studies may still have missing data, even when specific interventions to minimize missing data are used [7], particularly in elderly respondents [9, 12]. In the event of small, random amounts of missing data, a simple approach of replacing the missing values with the mean of the sample does appear to provide an adequate and practical solution that may be considerably more palatable for the average nurse researcher than the use of costly or complex alternatives.

## Figures and Tables

**Table 1:** Mean values for imputed and sample data.

Data source	PCS mean (SD)	MCS mean (SD)
Missing data estimation (*n* = 21)*	37.9 (11.5)	47.7 (10.3)
Assignment of mean score (*n* = 21)*	38.3 (10.1)	47.8 (10.4)
Difference (AMS-MDE)	0.35 (1.09)	0.16 (1.14)
Sample without imputed data (*n* = 384)	39.2 (9.9)	51.6 (9.8)
Sample with missing data estimation (*n* = 405)	39.1 (10.0)	51.4 (9.9)
Sample with assignment of mean score (*n* = 405)	39.1 (9.9)	51.4 (9.9)

PCS: Physical Component Summary; MCS: Mental Component Summary; SD: standard deviation; AMS: assignment of mean score; MDE: missing data estimation.

*Intraclass correlation between the two imputed PCS scores was  .991, *P* < 0.0001; between the two MCS scores it was  .997, *P* < 0.0001.

## Abbreviations

HRQOL: Health-related quality of life
PCS: Physical Component Summary
MCS: Mental Component Summary
MDE: Missing data estimation
AMS: Assignment of mean score.
